# Temporal classification of short time series data

**DOI:** 10.1186/s12859-024-05636-6

**Published:** 2024-01-17

**Authors:** Benedikt Venn, Thomas Leifeld, Ping Zhang, Timo Mühlhaus

**Affiliations:** 1 Computational Systems Biology, RPTU Kaiserslautern, 67663 Kaiserslautern, Germany; 2 Institute of Automatic Control, RPTU Kaiserslautern, 67663 Kaiserslautern, Germany

**Keywords:** Time series analysis, Smoothing spline, Profile classification, Omics analyis

## Abstract

**Motivation:**

Within the frame of their genetic capacity, organisms are able to modify their molecular state to cope with changing environmental conditions or induced genetic disposition. As high throughput methods are becoming increasingly affordable, time series analysis techniques are applied frequently to study the complex dynamic interplay between genes, proteins, and metabolites at the physiological and molecular level. Common analysis approaches fail to simultaneously include (i) information about the replicate variance and (ii) the limited number of responses/shapes that a biological system is typically able to take.

**Results:**

We present a novel approach to model and classify short time series signals, conceptually based on a classical time series analysis, where the dependency of the consecutive time points is exploited. Constrained spline regression with automated model selection separates between noise and signal under the assumption that highly frequent changes are less likely to occur, simultaneously preserving information about the detected variance. This enables a more precise representation of the measured information and improves temporal classification in order to identify biologically interpretable correlations among the data.

**Availability and implementation:**

An open source F# implementation of the presented method and documentation of its usage is freely available in the *TempClass* repository, https://github.com/CSBiology/TempClass  [58].

**Supplementary Information:**

The online version contains supplementary material available at 10.1186/s12859-024-05636-6.

## Introduction

Biological systems are constantly regulating their genes, proteins, and metabolites to maintain an optimal internal state. Optimal, however, is context-dependent and contingent upon prevailing environmental factors. Disturbances such as alterations in light, temperature, moisture, or mineral concentrations necessitate metabolic adjustments to mitigate potential stress and restore optimal conditions according to the external influence. These acclimation responses are meticulously orchestrated and follow a defined sequence. Their primary objectives are to mitigate the adverse effects of unfavourable environmental conditions or optimally exploit positive alterations.

With the declining costs associated with high throughput technologies like RNA-Seq and MS proteomics, the utilization of time series analyses has gained popularity as a valuable tool to study the kinetics/temporal dynamics of biological molecules. Nonetheless, challenges complicate comprehensive analyses of such data. Time series datasets often comprise a limited number of measurement points, and due to the substantial investment required in growing biological material and the still relatively high costs of these analyses, experiments are typically designed with a modest number of measurement points (typically 4 to 8) and a few replicates (2–4).

When characterizing the cellular response characteristics for individual molecules, it is imperative to assign lower significance to measurement points characterized by elevated uncertainty [1]. While this assignment is often intuitive when performed manually, an automated evaluation method necessitates the explicit incorporation of this consideration.

Simultaneously, the biological response capacity is constrained. High amplitude fluctuations are improbable from a regulatory standpoint, and they would entail substantial synthesis and degradation costs for the biological system in question. In the absence of new stimuli, one can reasonably anticipate smooth kinetics in biological molecules, especially for more complex molecules like proteins. This assumption provides valuable additional information that enhances the precision and utility of biological models. However, it mandates the development of novel analytical techniques to effectively incorporate such information.

Our proposed approach addresses the dual challenges of variable measurement uncertainties and the expectation of low signal fluctuation. We have employed smoothing splines, which impose continuity in function, slope, and curvature, while also permitting the weighting of individual measurement points and the imposition of shape constraints. Unlike existing methods that use predefined profiles [2], or require a preselected number of clusters [3], our approach classifies the data where a single classification is uncoupled from the remaining data.

## Methods

Time series data can be assumed as functions of time with superimposed heteroscedastic biological variance and technical noise [4].

$${y}_{i}=f\left({t}_{i}\right)+ {\varepsilon }_{i}, i=1,\dots ,n$$

where f(·) resembles the function of the true abundance time course at the ith of n time points. The error term ${\varepsilon }_{i}$ combines biological variation and noise introduced by sample processing and measurement devices, leading to the blurring of the true relationship f(·) to the final reading $y$ at time point $i$. The interval widths between measuring time points are defined by:

$${h}_{i}={y}_{i+1}-{y}_{i}, i=1,\dots ,n-1$$

While in many analysis strategies, e.g. common clustering procedures or statistical testing frameworks, interval widths are not taken into account, they may possess valuable information regarding the dynamic of the underlying kinetic [5, 6]. High amplitude changes within a short time period can be considered unlikely and thus penalized by the model. However, for biological regulatory responses, the model time point spacing may vary from the actual experimental time point spacing as discussed below.

### Time point spacing

As indicated by its name, the independent variable is time (e.g. hours since experiment start). For experiments with no perturbation, these time intervals can be directly used for curve fitting. Cell cycle regulation may be measured with fixed time intervals because the expected rate of change is evenly distributed between the time points. If the biological system, however, faces sudden condition perturbation, the spacing according to time intervals is insufficient for modelling. A perturbation causes the biological system to react immediately. This regulation of molecular processes has to occur quickly with regulation in later time points being less fluctuating. To account for this asymmetric regulation, samples are taken according to the expected rate of system response. The measurements of the presented experiment were taken by doubling the time interval at each measurement. This is according to the estimated change apparent within two measurements. Hence, samples can be spaced uniformly in time. The presented approach is not restricted to uniformly spaced time points and works with any univariate time series.

### Smoothing spline

To investigate the underlying kinetic, the application of smoothing splines offers a valuable approach to model the data, striking a balance between data fidelity and the smoothness of the fit. While various other fitting techniques exist, i.e. interpolation strategies or (non-) linear regression, most of them rely on a predefined template function or ignore point uncertainty as they are forced to interpolate the sample means. Further explanations for choosing smoothing splines over other fitting techniques are given in the discussion. In this method, piecewise cubic polynomials are employed to model each subinterval of the data while smooth transitions are ensured by enforcing the equality of function values, slopes, and curvatures at each designated knot. Considering that knots are positioned at each time point, there are $n-1$ intervals to analyze.

Splines within the interval $\left[{t}_{i},{t}_{i+1}\right]$ are defined as

1 $${f}_{i}\left(t\right)= {a}_{i}{\phi }_{0i}t+{a}_{i+1}{\phi }_{1i}t+{c}_{i}{\gamma }_{0i}t+{c}_{i+1}{\gamma }_{1i}t$$

with ${a}_{i}={f}_{i}\left({t}_{i}\right)$, ${c}_{i}={{f}^{{\prime\prime}}}_{i}\left({t}_{i}\right)$, and basis functions ${\phi }_{0}$, ${\phi }_{1}$, ${\gamma }_{0}$, and ${\gamma }_{1}$ defined in [7] and listed in the supplement. The vector $a$ is the vector that only contains the function values at the measured time points, $c$ contains the function curvature (second derivative) at the measured time points and the basis functions describe how the adjacent knots influence the curve shape between them.

The estimation of spline segments is subject to the minimization of the following cost function [7, 8]:

2 $$\underset{{t}_{1}}{\overset{{t}_{n}}{\int }}{\left[{f}^{{\prime}{\prime}}\left(t\right)\right]}^{2}dt + \frac{\lambda }{n}{\Vert W(a-y)\Vert }^{2}, {W}_{i,i}={w}_{i}$$

$W$ is a diagonal matrix of observation weights introduced in Eq. 10, $a$ is a rowvector of the splines function values at the knots, $y$ is a rowvector of the observation values, and || · || denotes the Euclidean norm. While the first term serves as a roughness penalty, the second term ensures the required fidelity to the data [9]. A smoothing factor $\lambda$ mediates between these opposing error terms. When $\lambda =0$, the resulting spline results in a straight least squares regression line, while $\lambda \to \infty$ leads to an interpolating cubic spline.

In-depth spline theory is given in [8–12]. The minimization of Eq. 2 can be rewritten as a quadratic optimization problem [7].

3 $$\substack{min\\a} \frac{1}{2}a^{T} G_{\lambda } a + c_{\lambda }^{T} a,$$

with ${G}_{\lambda }=2\left({H}^{T}{D}^{-1}H+ \frac{\lambda }{n}{W}^{T}W\right)$ and ${c}_{\lambda }=-2\frac{\lambda }{n}{y}^{T}{W}^{T}W$. Band matrices $D$ and $H$ are defined as:

4 $${D}_{n-2 \times n-2}=\left[\begin{array}{cccccccc}{d}_{1}^{a}& {d}_{2}^{b}& 0& 0& \cdots & 0& 0& 0\\ {d}_{2}^{b}& {d}_{2}^{a}& {d}_{3}^{b}& 0& \cdots & 0& 0& 0\\ 0& {d}_{3}^{b}& {d}_{3}^{a}& {d}_{4}^{b}& \cdots & 0& 0& 0\\ \vdots & \vdots & \vdots & \vdots & \ddots & \vdots & \vdots & \vdots \\ 0& 0& 0& 0& \dots & {d}_{n-3}^{b}& {d}_{n-3}^{a}& {d}_{n-2}^{b}\\ 0& 0& 0& 0& \cdots & 0& {d}_{n-2}^{b}& {d}_{n-2}^{a}\end{array}\right]$$

5 $${d}_{i}^{a}={(h}_{i}+{h}_{i+1}) / 3$$

6 $${d}_{i}^{b}={h}_{i} / 6$$

7 $${H}_{n-2 \times n}=\left[\begin{array}{cccccccc}{e}_{1}^{b}& {e}_{1}^{a}& {e}_{2}^{b}& 0& \dots & 0& 0& 0\\ 0& {e}_{2}^{b}& {e}_{2}^{a}& {e}_{3}^{b}& \dots & 0& 0& 0\\ \vdots & \vdots & \vdots & \vdots & \ddots & \vdots & \vdots & \vdots \\ 0& 0& 0& 0& \dots & {e}_{n-2}^{b}& {e}_{n-2}^{a}& {e}_{n-1}^{b}\end{array}\right]$$

8 $${e}_{i}^{a}={-(h}_{i}+{h}_{i+1}) / ({h}_{i}\cdot {h}_{i+1})$$

9 $${e}_{i}^{b}=1 / {h}_{i}$$

### Measurement weighting

A crucial part of spline smoothing is the determination of the weighting matrix $W$. For each signal, the time point weighting ${w}_{i}$ relies on the signal’s standard deviation that is divided by the average standard deviation of all time points. As smoothing splines—under the given smoothness constraints—aim to minimize the distance of the original data points to the resulting prediction (sum of squares), outlier values would negatively impact the prediction function. As time points with outliers often are affected by high uncertainty, this variance can be exploited to reduce the outlier impact. When calculating the sum of squares, high variances are encoded as low weights that reduce the impact of points, which should have reduced influence on the fit (Eq. 2).

10 $$W=\left[\begin{array}{ccccc}{w}_{1}& \cdots & 0& \cdots & 0\\ \vdots & \ddots & \vdots & \ddots & \vdots \\ 0& \cdots & {w}_{i}& \cdots & 0\\ \vdots & \ddots & \vdots & \ddots & \vdots \\ 0& \cdots & 0& \cdots & {w}_{n}\end{array}\right], {w}_{i}=\frac{{w}_{init}\left(i\right)}{\overline{{w}_{init}}}$$

11 $${w}_{init}(i)={ \sigma }_{{y}_{i}}$$

### Shape constraints

To control the spline’s shape, monotonicity can be enforced for each interval separately. To obtain a single maximum within interval ${h}_{i}$, combinations of monotonicity constraints are applied, so that intervals within $[{t}_{0},{t}_{i-1}]$ are monotonically increasing, and intervals in $[{t}_{i+1},{t}_{n}]$ are monotonically decreasing. Monotonicity constraints are well studied and can be enforced by the following conditions [13–16].

For monotonically increasing polynomials in $\left[{t}_{i},{t}_{i+1}\right]$ ensure that

12 $${a}_{i+1}-{a}_{i}\ge 0$$

13 $${{f}^{\prime}}_{i}\ge 0$$

14 $${{f}^{\prime}}_{i+1}\ge 0$$

15 $$3\frac{{a}_{i+1}-{a}_{i}}{{h}_{i}}-{f^{\prime}}_{i}\ge 0$$

16 $$3\frac{{a}_{i+1}-{a}_{i}}{{h}_{i}}-{f^{\prime}}_{i}\ge 0$$

For monotonically decreasing polynomials signs are switched from $\ge$ to $\le$. The derivative ${{f}^{\prime}}_{i}$ is calculated from ${B}_{i}a$ where $B$ is a $n \times { }n$ Matrix defined by $B = P^{ - 1} U$. *P* and $U$ are described in [7] and can be seen in the supplement. The conditions for every interval are summarized in a constraint matrix $Ca\ge [0]$ representing linear inequality constraints. When solving (12–16) with respect to $Ca\ge \left[0\right]$ some constraints will be satisfied as equality constraints. This constraint set where $C_{A}a=\left[0\right]$ is termed ‘Active set’. Matrix $Z$ which columns form a basis for the null space of ${C}_{A}$ is used for determining the smoothing factor $\lambda$ [7, 17]. Using smoothing splines in combination with the described monotonicity constraints allows the construction of smooth curves with oscillations allowed in specified regions that are not constrained to be monotone.

For every signal to fit, several monotonicity constraints can be applied, resulting in a range of potential parent shapes. These parent shapes span a spectrum from monotonically in- or decreasing curves to those featuring 1, 2, 3, or 4 extrema, with each type starting either with a maximum or a minimum. This results in a total of 10 distinct parent shapes, along with a single unconstrained scenario, that can be applied to a single time series signal.

Under the specified slope constraints, the case of monotonically increasing or decreasing curves offers only one shape, while curves containing at least one extremum necessitate the consideration of multiple shape possibilities (as summarized in Additional file 1: Table S1). Consequently, it is necessary to fit curves corresponding to all conceivable shapes to each signal. Subsequently, the most appropriate shape is selected as the descriptor for elucidating the underlying molecule kinetics.

### Model selection

Besides the data points with an associated weighting matrix, smoothing splines rely on a smoothness parameter $\lambda$ that controls how curved the resulting curve is going to be. The smoothing strength must be determined individually for each signal. The ideal $\lambda$ value is estimated by minimizing the modified generalized cross validation (mGCV), which is an estimate for the model prediction error and—in contrast to other cross validation techniques—only requires a single passage. A minimal mGCV hints at the optimal compromise between over- and underfitting respectively [12, 18, 19].

17 $$\begin{array}{c}\mathit{min}\\ \lambda \end{array} n\frac{{\Vert W(a-y)\Vert }^{2}}{{\left(Tr(I-{\rho A}_{\lambda })\right)}^{2}}$$

Matrix *I* is a $n\times n$ identity matrix and ${A}_{\lambda }=2\frac{\lambda }{n}Z{({Z}^{T}{G}_{\lambda }Z)}^{-1}{Z}^{T}{W}^{T}W$ an influence matrix, so that $a=Ay$ and $\rho =1.3$ as compensatory factor for small sample sizes [7, 8, 20]. Because the minimization of Eq. 17 with respect to Eq. 3 leads to a non-convex and non-continuous optimization problem, a grid search approach is used to choose from a wide range of $\lambda$.

For every parent shape, a single candidate is reported and selected by minimizing mGCV. When a maximum of four extrema is allowed, this results in 11 final fits to choose from ((i) 5 fits with 0–4 extrema starting with a positive slope, (ii) 5 fits with 0–4 extrema starting with a negative slope, and (iii) an unconstrained spline that has no assumptions regarding its monotonicity). While mGCV proves suitable for making reasonable determinations of the smoothing strength within the parent shape class, it encounters challenges when confronted with the task of selecting the correct shape from the remaining 11 shape options. As mGCV does not include information about the number of allowed extrema it tends to favour fits with increased flexibility over conservative ones.

An adapted version of the Akaike information criterion (AIC) is used to select the final shape [21]. This modified approach incorporates a correction factor tailored for cases with limited sample sizes (AICc) as originally proposed in [22] and a term that incorporates the number of extrema present in the curve.

18 $${AIC}_{c}=n\cdot {\text{ln}}\left(\frac{{\Vert W\left(a-y\right)\Vert }^{2}}{n}\right)+2k+\frac{2{k}^{2}+2k}{n-k-1}$$

Here $k$ equals the enforced number of extrema.

Among all shape assumptions, the model that minimizes ${AIC}_{c}$ (Eq. 18) is assumed to represent the underlying function at best and is used for temporal classification.

### Extrema extraction and classification

It is trivial to identify the splines extrema, since all polynomial coefficients can easily be obtained for every interval from function values $a$ and their second derivatives $c$ at adjacent knots. Polynomial template:

19 $$s(t) = k_{1} t^{3} + k_{2} t^{2} + k_{3} t + k_{4} ,$$

with $$s\left({t}_{i}\right)={a}_{i}$$, $$s\left({t}_{i+1}\right)={a}_{i+1}$$, $$s^{\prime\prime}({t}_{i})={c}_{i}$$, and $${s}^{{\prime}{\prime} }({t}_{i+1})={c}_{i+1}$$

Basic calculus leads to the polynomial coefficients:

20 $${k}_{1}=-\frac{1}{6}\left({c}_{i}+{c}_{i+1}\right)$$

21 $${k}_{2}=\frac{1}{2}{c}_{i}$$

22 $${k}_{3}=-\frac{1}{3}{c}_{i}-\frac{1}{6}{c}_{i+1}-{a}_{i}+{a}_{i+1}$$

23 $${k}_{4}={a}_{i}$$

Extreme points are determined by setting ${s}^{\prime}\left(t\right)=0$ and ${s^{\prime\prime}}\left(t\right)<0$ for maxima and ${s^{\prime\prime}}\left(x\right)>0$ for minima within $\left[{t}_{i},{t}_{i+1}\right]$. Additional file 1: Fig. S6 gives a visual impression of the extrema extraction process. The location of extreme points is used to group similar shaped time series. Although the location of extreme values is determined by the model, it is necessary to determine the exact position after a model is fitted to the data. This downstream identification of extrema enables previous filtering of quasi-constant signals and ensures that extrema can be assigned to their nearest knots. The classifier may result in `Min3,Max4` indicating the spline having a minimum at the third time point and a maximum at the fourth time point. If there is no extremum, the classifier results in either `I` or `D`, depending on whether the spline is monotonically in- or decreasing. If necessary, these two monotone classes may be further refined using the second derivative to identify prominent changes in curvature (plateau regions).

### Iterative clustering

As a common time-series clustering procedure, $k$-means clustering is used and compared to the presented classification method. The $k$-means algorithm iteratively recalculates the position of $k$ initial centroids. All points are assigned to the nearest centroid, while the squared Euclidean distance is used as distance measurement. Convergence is reached when no reallocation of points to different centroids occurs [23, 24].

The optimal cluster number $k$ is determined by the gap statistics method [25].

If relative changes of two signal slopes behave the same but their absolute values differ, most distance measures would report high distances even if their parallel change would suggest a difference of 0 (or similarity of 1). To prevent dominance of variables with differing ranges a normalization step often is needed to equalize all amplitudes. A popular normalization is the z-score, which transforms a single time series to have zero mean and unit variance by

24 $${y^{\prime}}_{k}=\frac{{y}_{k}-\mu }{\sigma }$$

when $\mu$ denotes the arithmetic mean and $\sigma$ is the standard deviation of the given data ${y}_{k}$[26]. This analysis was performed using FSharp.Stats v0.5.0 [27].

### Comparison of leave one out cross validation

In order to assess the robustness of the used constrained smoothing spline, protein time series were fitted using four different curve fitting methods: (i) constrained smoothing spline as presented in this work, (ii) polynomial interpolation of sample averages, (iii) linear spline interpolation of sample averages, (iv) cubic spline interpolation of sample averages. All but the constrained smoothing spline procedures are available at FSharp.Stats v0.5.0. For leave one out cross correlation all three replicates of an internal sample were deleted from the time series.

After deletion all four fitting procedures were applied to the modified time series. The distance of the prediction at the time point of missing data to the original prediction is determined. For each protein signal, this leads to 6 distances. The same procedure was applied using the distance from the prediction at the time point of missing data to the sample mean (Additional file 1: Figure S1).

### Visualization

All visualizations presented in this manuscript were prepared using Plotly.NET v4.0.0 [28].

### Enrichment analysis

Gene set enrichment analysis is performed using extended MapMan annotations (for example "PS.lightreaction.LHC" becomes "PS.lightreaction.LHC", "PS.lightreaction" and "PS") [29–31]. All 1292 proteins served as background if overrepresentation of functional annotations is studied within classes. Enrichment was performed using multiple hypergeometric tests while p values were corrected for multiple testing using Storey’s q value method [32, 33].

## Results

**Fig. 1:**
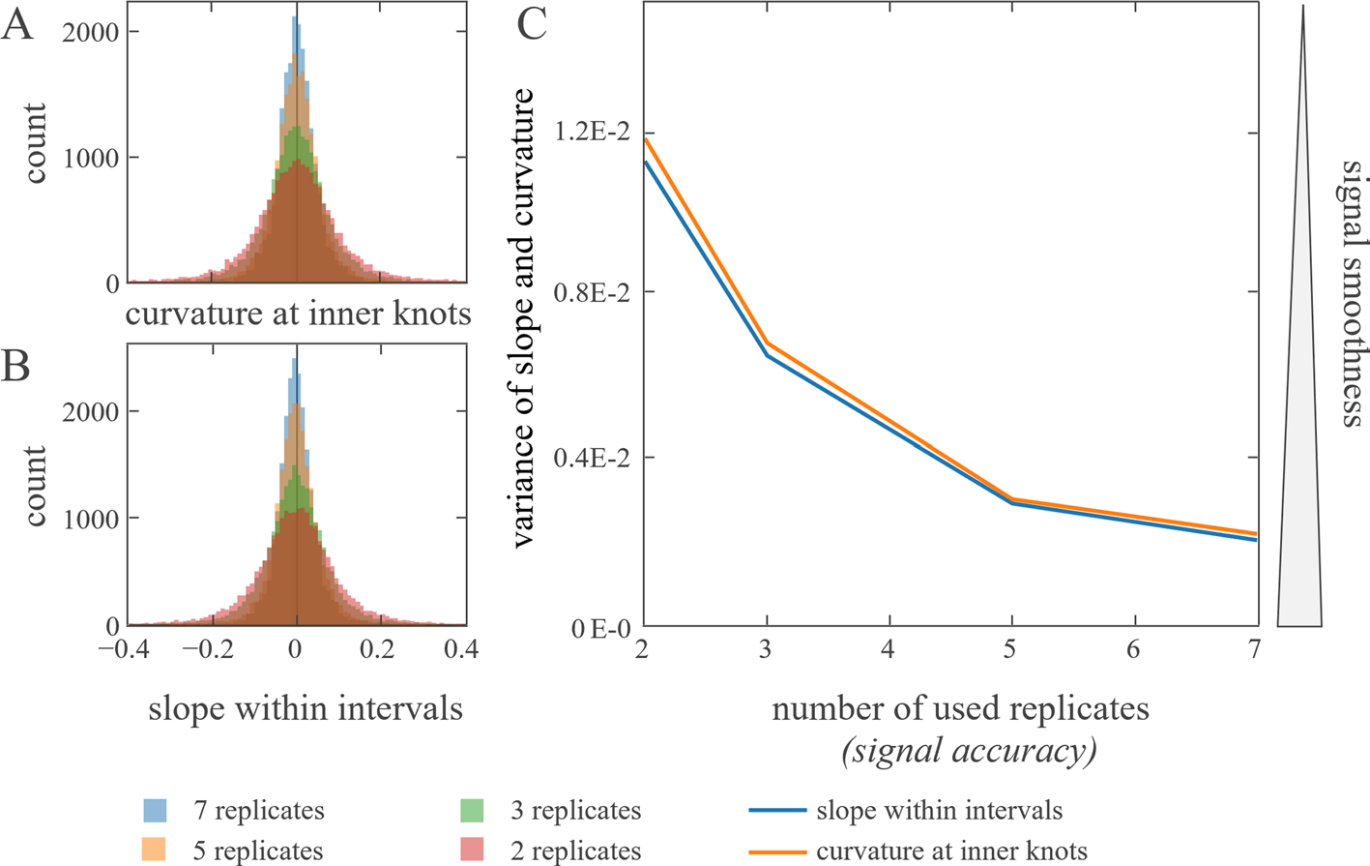
In a circadian time series proteomics experiment, protein intensities were measured every four hours for two days (PXD019431). To investigate the smoothness of the signal, replicates at each time point were shuffled randomly and reduced to a replicate number given in the legend. In each data reduction iteration curvature and slopes were determined. **A**: Protein intensity means at 12 time points were interpolated with cubic splines using natural boundary conditions [27]. The second derivative of the spline at the inner knots was determined as smoothness measure. **B**: Protein intensity means at 12 time points were interpolated with linear splines. The first derivative of the lines within the knot intervals was determined as smoothness measure. **C**: Variances of curvatures **A** and slopes **B** of the signal interpolation are calculated and plotted against the number of used replicates

Our approach assumes biological molecule kinetics have the intrinsic constraint to avoid curvature and therefore be smooth. To underpin this assumption with data, a time series dataset with 12 time points was analysed, which were analysed with 7 replicates each. Naturally, the more replicates measured, the more accurate the abundance estimator is supposed to be. To validate the smoothness assumption 2, 3, 5, and 7 replicates of each protein were randomly selected and analysed. The signals were interpolated with both linear splines and cubic splines, and the slope (linear spline) and curvature (cubic spline) at each knot were extracted. Both slopes and curvatures of the signals decrease with increasing number of replicates (Fig. 1). The higher the measurement accuracy (increased number of replicates), the higher the signals smoothness (reduced variance in slope and curvature) supporting the assumption of biological molecule kinetics showing the tendency of being smooth.

**Fig. 2:**
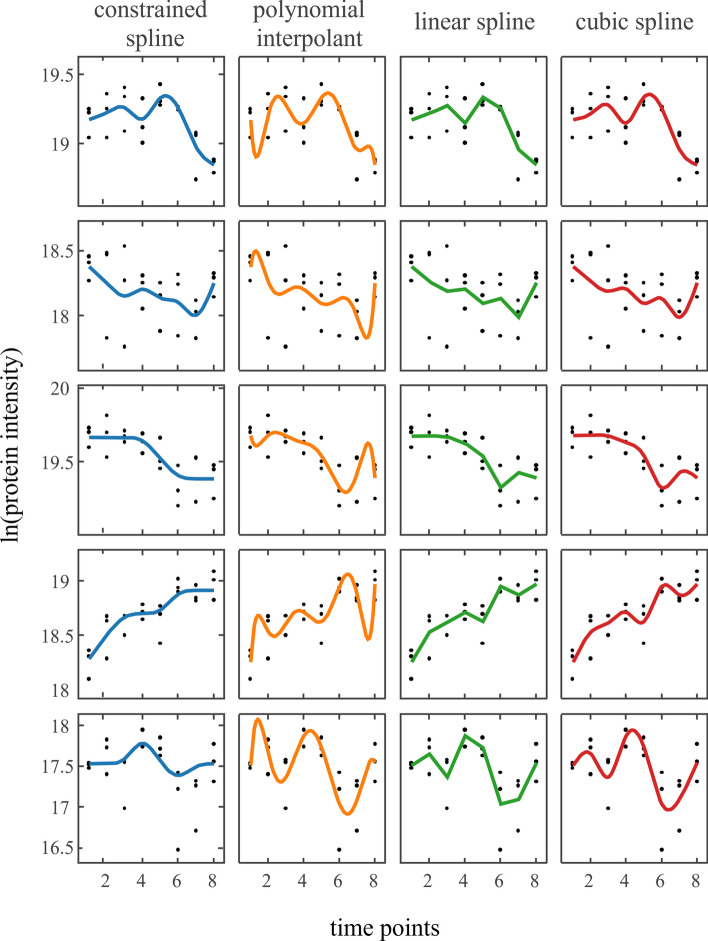
Four curve fitting approaches on five proteins. Five exemplary protein abundance signals (top to bottom) modelled using four approaches: (i) constrained smoothing spline, (ii) polynomial interpolation of arithmetic means, (iii) linear spline interpolation of arithmetic means, and (iv) cubic interpolating splines with natural boundary conditions

Based on the constrained smoothing splines, we classified protein abundances from a heat acclimation experiment conducted in the green algae *Chlamydomonas reinhardtii* [34]. The utilized data subset consists of measurements taken at 8 time points during 40 °C heat treatment (0 h, 0.5 h, 1 h, 2 h, 4 h, 8 h, 16 h, and 24 h respectively). 1,292 proteins are measured in three biological replicates. An exemplary comparison of four fitting methods is given in Fig. 2. Constrained smoothing splines ensure smooth curves while being flexible enough to recognize relevant changes.

The signals were classified by the location of their extrema as described in *Extrema extraction and classification*. For 8 measured time points, 327 possible curve configuration possibilities (shapes) exist (Additional file 1: Table S1).

**Fig. 3:**
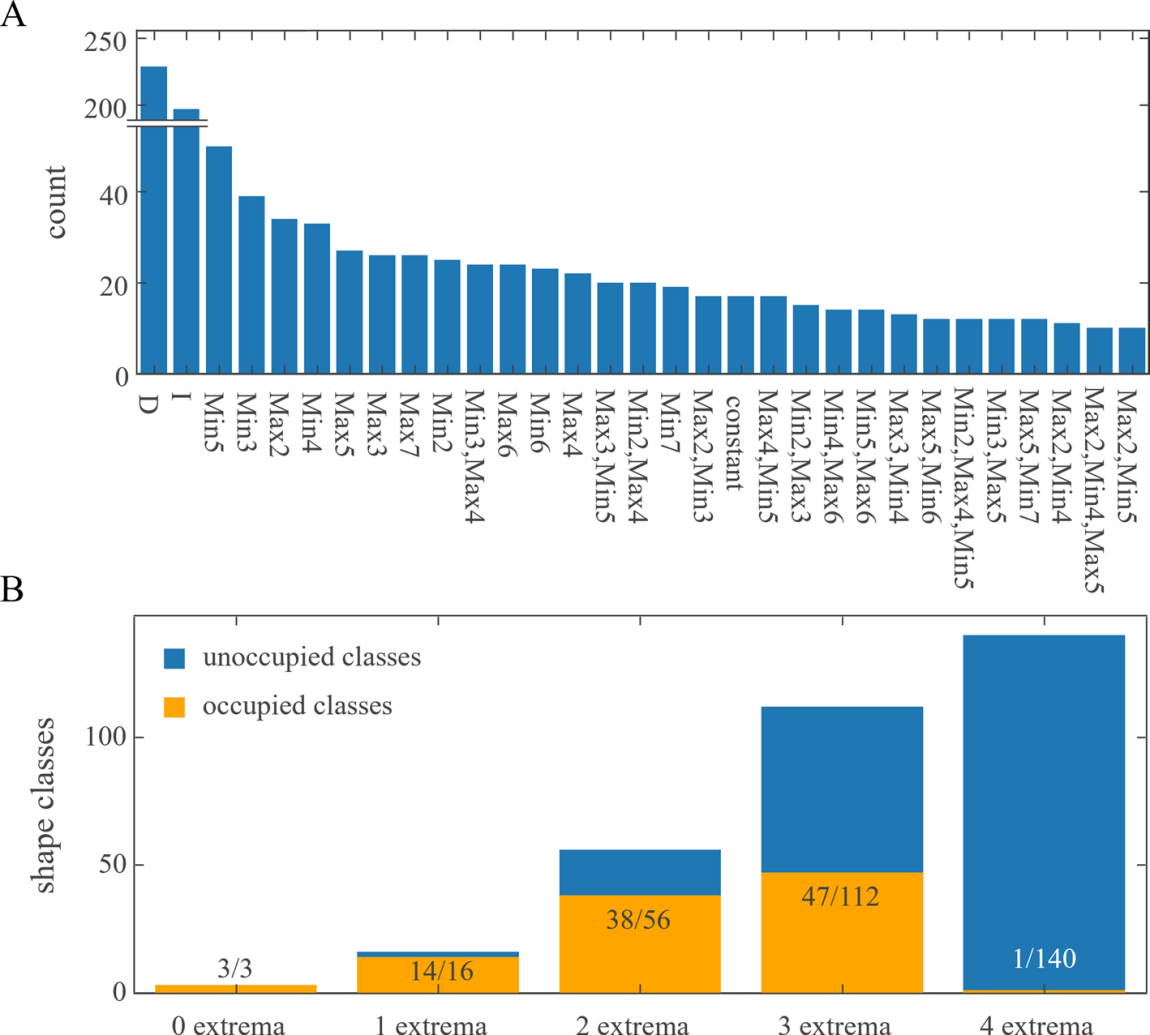
Class occupancy **A** All classes that had more than 10 proteins assigned are depicted. Min3 indicates a single minimum at the third time point. Min3, Max4 indicates a minimum at the third time point followed by a maximum at the fourth time point. **B** Number of possible classes (blue) and number of occupied classes in the presented data set. No extremum is present in constant, monotonically in- and decreasing signals

The analysis of the smoothed protein signals resulted in 77 classes to be filled with at least one protein signal. As expected, the classes become smaller with increasing specificity (Fig. 3). Classes with a high number of extreme points must have strong evidence of such behaviour in the form of low variances and high amplitude differences. The smoothness constraint therefore leads to less class occupancies the more complex the class gets, despite the fact, that the shape possibilities increase with more extrema (Fig. 3B).

### Determination of fit robustness

**Fig. 4:**
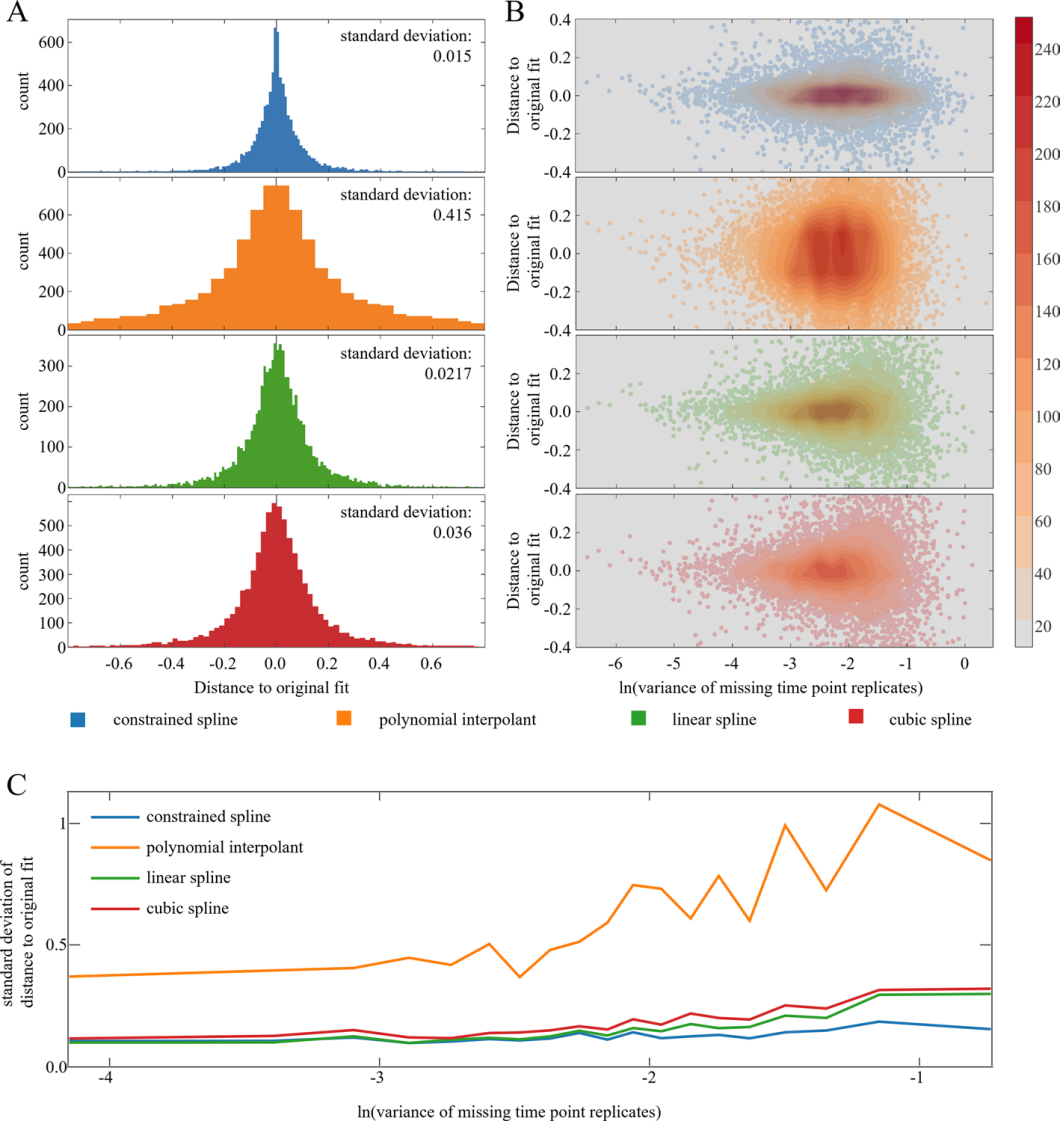
Robustness analysis. **A**: Four fitting techniques were applied to each protein signal. After deleting every inner time point once, the distance from the original prediction to the prediction using the sparse signal is measured. For each protein, 6 distances are reported (number of inner time points) and summarized in a histogram. The histogram’s standard deviation is given in the top right. **B**: The same data was used as in **A** but additionally separated by the variance of the time point replicates that were deleted. **C**: The distance data is separated in 20 equally large bins depending on the variance of the missing time point replicates (**B** x axis). The standard deviation of the distances within each bin is calculated and plotted against the average time point variance

To compare the use of constrained smoothing splines with other fitting methods, the data were cross-validated as explained in *Comparison of leave one out cross validation*. All replicates of an inner time point were removed, and the remaining time points fitted with the presented constrained smoothing spline, interpolating polynomial, linear spline, and cubic spline. The distance of the predicted value at the time of the missing data to the original prediction serves as a robustness measure. If a data point is deleted from the time series, the model curve may change in shape. The higher this change, the more prone to overfitting a model is. Simultaneously high distances indicate a high influence of the particular point for the model. The protein signals range from 16 to 24 with a median standard deviation of 0.156. As expected, polynomial interpolation leads to massive overfitting (compare curve shapes in Fig. 2). With increasing variance at the point of interest, the overfitting tendencies of linear and cubic splines tend to increase (Fig. 4B, C).

Especially when variance is high in the missing time point replicates, the constrained spline assigned lower weightings to this point and shows a reduced distance to the original curve (Fig. 4). To examine whether this robustness is solely due to a conservative fitting, the same procedure was performed using the distance of the prediction of the sparse data at the time point of missing replicates to the original sample mean instead of the original prediction. Low distances in this measure would hint at underfitting, indicating that the model isn’t at all influenced by the signal manipulation. Because the polynomial as well as the linear and cubic spline interpolates the mean, the distances stay the same. The constrained spline shows similar distances as the other fitting techniques, indicating a comparable fidelity to the data and not giving suspicion for underfitting tendencies (Additional file 1: Fig. S2).

### Comparison to clustering approaches

Besides statistical methods, clustering approaches are the most common analyses of biological time series and thus are a genuine reference for our approach.

For this purpose, the signals should be transformed in advance so that they have zero mean and unit variance. However, signals whose abundance does not change would be strongly distorted by this transformation. In order to increase the clustering quality, such signals can be filtered out. A simple to use quality filtering approach often seen on biological time series data is the application of a one-way ANOVA. Its main purpose in clustering approaches is not the detection of significances, but to filter signals whose average did not change during the time course. As a common threshold a p value of 0.05 was chosen. Note that the presented method of temporal classification does not require this step, since signal classification is not affected by the presence of other signals.

**Fig. 5:**
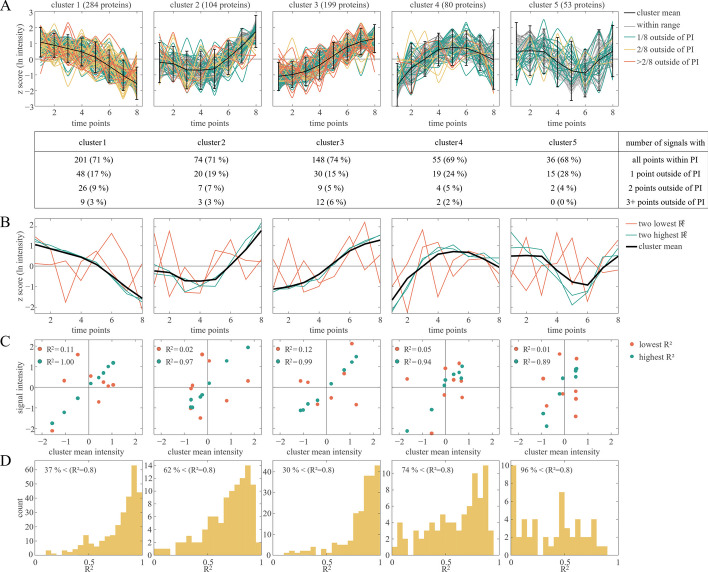
Iterative clustering result. 720 protein signals were individually transformed to z scores and subsequently clustered by k means clustering (*k* = 5). **A**: Five clusters are depicted with cluster mean and 95% prediction interval (PI). Signal colours indicate whether 0 (grey), 1 (green), 2 (yellow), or more (orange) points of a signal lie outside of the PI. The table below shows the percentage of each group. **B**: The cluster mean intensity is visualized together with signals that showed the highest (green) and lowest (red) coefficient of determination (R^2^) to the cluster mean. **C**: The cluster mean intensity is plotted against the intensities of the signal of highest (green) and lowest (red) R^2^ within the cluster. R^2^ of both signals is given in each panel. **D**: Histogram of all R^2^ values between signals and cluster mean. The percentage in each panel depicts the percentage of signals whose R^2^ is lower than 0.8

Clustering of the remaining 720 protein signals was performed using the k means algorithm with Euclidean distance [27]. The optimal number of clusters was determined to be 5 (Additional file 1: Figure S3). It is obvious that five clusters do not sufficiently represent all regulatory responses within a biological system. It is a suitable methodology for obtaining a global impression of the data set and a summary of the major protein kinetic groups. But for a detailed description of the response of fine-tuned biological processes, this approach is too rough. Interesting regulatory details are blurred by the sheer amount of data within a single cluster (Fig. 5, Additional file 1: Fig. S4). Regression analysis reveals that for every cluster 30% of the protein signals are not within the 95% prediction interval of the cluster mean. If the regulatory response of proteins is studied, in at least 30% of the cases a classification based on the cluster would be inaccurate at best.

### Biological interpretation

Besides using the smoothed signals for comparative analysis (e.g. co-expression networks), the smoothed and classified protein signals can be used for exploratory data analysis. To elucidate early, but short-term responders of the heat treatment, signals of the classes “Maximum at 2 or 3" can be isolated and used for categorizing the acclimation response.

Furthermore, global analysis strategies can be applied to classified signals. Gene set ontology enrichments of molecular functions can be applied to identify function overrepresentation.

**Fig. 6:**
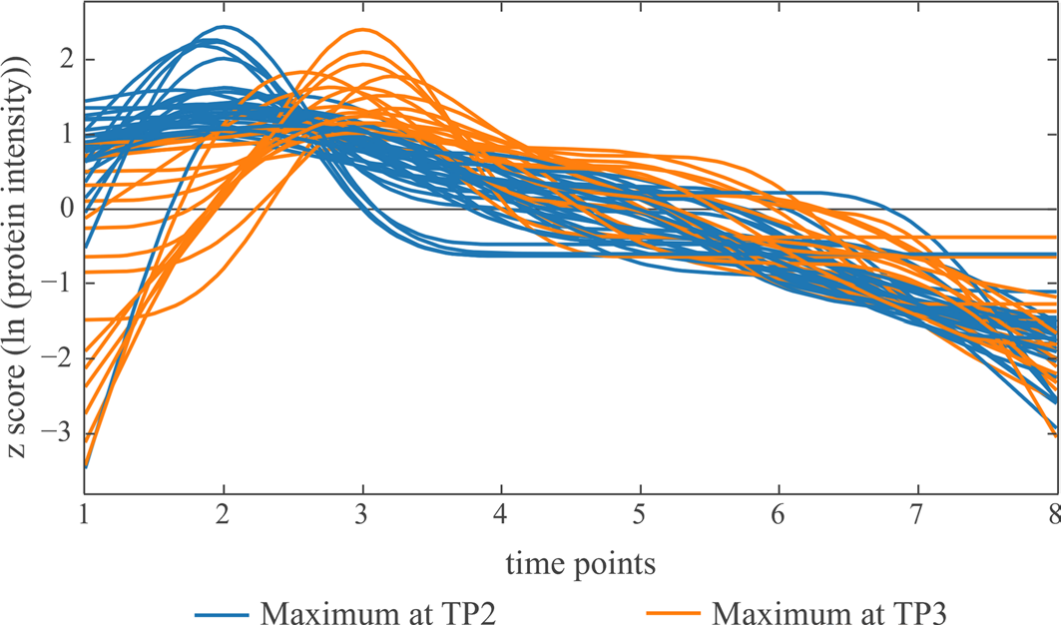
Visualization of two classes that show a single maximum at time points 2 or 3 respectively. The intensity signals of 50 proteins are transformed to have zero mean and unit variance

Obviously, due to the sensitivity of the classification, the number of classes is by far greater than the number of clusters using common clustering strategies. This results in sparse occupancy of shape classes, which impedes enrichment strategies. However, the possibility of subsequent class aggregation presents valuable opportunities for analysing different combinations of response types. A gene set enrichment analysis was conducted on the early responder classes (Fig. 6) using MapMan functional annotations for *Chlamydomonas reinhardtii* genome version 5.5 [29, 30, 32]. Functional annotations that were overrepresented within the early responders can be seen in Table 1.

**Table 1:** Enrichment result of early responder class

Functional term	q value	Trivial names (if annotated)
Protein.synthesis.ribosomal protein	0.0164	rps9; PRPL1; MRPL1; RPL23A; UBQ2; RPL40; UBQ1; RPS7; RPL18; RPL12; RPS27E1; RPL7; PRPL19;
Transport	0.0462	ATPVH;ATPVH;MPC1;AAA1;
Protein.synthesis.ribosomal protein.eukaryotic.60S subunit	0.0472	RPL23A; UBQ2; RPL40; UBQ1; RPL18; RPL12; RPL7
Protein.degradation.ubiquitin	0.0161	PKL1; UBC2; UBQ2; RPL40; UBQ1; UBQ2; RPL40; UBQ1; EIF3F; RPT4
RNA.RNA binding	0.0472	UBC2;REF1;HNR1
Transport.metabolite transporters at the mitochondrial membrane	0.0472	MPC1;AAA1
Polyamine metabolism.synthesis	0.0161	SPS1;SPD1

Functional annotations based on the MapMan ontology are listed together with the associated q value and trivial names if proteins had such

All functional annotations that are overrepresented in regulation shortly after heat onset were previously described to be involved in early heat acclimation regulation. (i) ribosomal proteins are required for the fast production of proteins; (ii) the transport group contains proteins predominantly involved meeting the increased demand of energy [35]; (iii) ubiquitin related proteins are necessary to both, degrade proteins that interfere with a heat acclimation, and remove proteins that aggregated due to the increased heat [36]; (iv) RNA binding proteins are involved in processing, stabilizing and exporting newly transcribed mRNA [37, 38]; (v) proteins of the polyamine synthesis group have been described to increase thermos-tolerance in algae [39].

The biological dissemination reveals that the classification approach is capable of elucidating the time-resolved orchestration of cellular responses and differentiating between different forms of regulation within a functional set of biological molecules.

## Discussion

The era of high throughput technologies enabled researchers to analyse the abundance of thousands of molecules in a time-resolved manner. Scientists once needed days to take samples and measure the signals of a few proteins individually. Nowadays, it is possible to quantify the entire transcriptome or proteome in one fell swoop. Although it is possible to measure the kinetics of hundreds of proteins at a time, the number of robust strategies for an analysis of temporally resolved cell responses remains small [40–42].

Clustering methods have always been a popular tool to analyse time series experiments, as these are great options for unsupervised methods that work well with only a few assumptions to be made. For example, k-means clustering of time point averages represents the most commonly used algorithm for unsupervised analysis as its computation is efficient and the resulting clusters can easily be interpreted [43–45].

Although great findings have been made by this approach [46–48], it poses two problems when used for temporal characterization of regulation responses: (i) Most commonly used distance measures consider each coordinate separately. The time series vectors can be shuffled in pairs and still obtain the same distance. This behaviour is contrary to biological intuition because transcript or protein quantities are strongly dependent on the previous time points. This dependence is not taken into account in the model and inevitably leads to a decrease in the quality of the signal-to-noise separation [49, 50]. (ii) Although there are also distance measures that act in an environment-dependent manner (Dynamic Time Warping), in clustering methods it is necessary to specify the number of clusters in advance. This reduces accuracy, since small definable groups may be sorted into large groups, and their identification is thus only possible manually. Numerous ways of determining the optimal number of clusters have been developed (Elbow criterion, Silhouette index, Gap statistics) [25, 51], but in most cases these underestimate the number of biological response forms present. Furthermore, clustering approaches often are used to subsequently classify the data based on features that are visible when looking at whole clusters, but not necessarily are valid for individual cluster elements [52, 53]. As shown, this is prone to result in a huge number of misclassifications (Fig. 5). These and other similarity-based techniques are not able to dissect delicate regulation responses, but instead, these signals might be blurred by averaging effects.

Our approach models the kinetic response by constrained smoothing splines with the incorporation of measurement variance. Several other fitting techniques can be applied, each of which addresses different assumptions. Linear splines are the least complex fitting model for time series as they just connect the point estimates (median or average) at each time point. Point weighting is not possible and there is no separation of signal and noise. For the same reasons, other interpolating methods such as interpolating polynomials or cubic splines are not suited.

An exception is a polynomial-based function approximation with Chebyshev knots. A major problem with interpolating polynomials is Runge's Phenomenon. This is manifested by high frequency oscillation of the function in the outer knot intervals [54]. By clever rearrangement of the knots away from the curve centre and towards the peripheral areas such an oscillation can be prevented [55]. At the same time, the function no longer passes through the original data points. However, disadvantages here are both the non-obvious selection of knots and their weighting, as well as the lack of methods to comply with monotonicity constraints. Linear or nonlinear regression techniques seem inappropriate as they require either (i) the selection of a polynomial degree that does not represent any meaningful biological interpretation, or (ii) an already predefined function that is fitted to the signal.

The modelling of the time series by constrained smoothing splines is based on smoothness assumptions and the consideration of measurement point variances. Additionally, this regression approach preserves the existing dependence between neighbouring time points and therefore enforces monotonicity where excessive oscillation is unlikely. When compared to other interpolation methods (polynomials, linear splines, cubic splines) our approach showed high robustness while being flexible enough to capture characteristic events during the time course (Figs. 2, 4). Due to the overfitting tendency and presence of Runge’s spike oscillation, polynomial interpolation is unsuited for classification analysis that handles extrema position as its primary characterization criterion. Linear splines show a high sensitivity for false declaration of extrema and perform poorly when it comes to predicting function values between the measured time points. Despite that cubic interpolating splines inherently aim to reduce heavy oscillations and perform great when it comes to predicting within intervals, their interpolating nature and inability to be weighted lead to little, but noticeable oscillations that interfere with an extrema-based classification strategy. While the number of shape classes can go to the hundreds, we could show that shapes with high flexibility and oscillations are found rarely (Fig. 3). This corresponds to the biological intuition of smooth protein regulation which was confirmed in Fig. 1.

Hybrid approaches are available that extend clustering approaches with prior smoothing regression [56], or by selecting meaningful expression profiles [1]. These approaches still rely on unsupervised approaches to group the data without predefining group labels. If faced with short time series data not exceeding 10 measurement time points, we propose that the number of group labels is manageable, hence all possible response shapes could be examined. However, a comparison of classification and clustering approaches remains difficult since the ground truth is unknown and both approaches address different questions. Most clustering approaches measure distances between signals, while classifications are concerned with the dissection of signal features. The incorporation of the information that biological signals tend to be smooth and not oscillating leads to a feature extraction that corresponds to the intuition regarding the regulation of biological molecules.

With our temporal classification approach for studying time resolved regulation, it is possible to not only find an optimal fit of the data, but also assign shape classes to large time series data sets. This makes it possible to analyse the temporal orchestration of acclimation response and actively search for patterns of interest. We were able to show that our method provides a robust estimator when faced with sparse data. Furthermore, a well-studied process of heat acclimation of *Chlamydomonas reinhardtii* was presented as an example of the method enabling a detailed and supervised analysis of specific acclimation responses.

The smoothing and classification algorithms can be accessed as F# implementation at https://github.com/CSBiology/TempClass (Additional file 1: Figure S5) [58].

### Limitations

This classification strategy is based on the selection of the optimal combination of monotone regions and extreme points. This approach requires an inner optimization to obtain the best fit of any enforced combination of monotonicity constraints, and an outer optimization to select the most ideal of the best shapes. With an increasing number of measured time points, there is a combinatorial explosion of the number of potential curve configurations. This not only increases the calculation time exponentially, but also the large number of resulting classes becomes unmanageable. Therefore, we have limited the number of allowed extreme points to 4 and recommend temporal classification for time series with 4 to 12 measurement time points.

### Supplementary Information

**Additional file 1:**  Spline basis functions, Table S1, and Figures S1 - S6.

## Data Availability

The data set the method was applied on is available in the repository: *Systems-wide investigation of responses to moderate and acute high temperatures in the green alga Chlamydomonas reinhardtii*, accessible at 10.60534/9e5jx-75d83 [57].
