# Supplementary material for: Temporal classification of short time series data
Source: BMC Bioinformatics. 2024 Jan 17;25:30. doi: 10.1186/s12859-024-05636-6 (PMC10792935; doi:10.1186/s12859-024-05636-6)
Supplement: Supplementary file 1 — Additional file 1: Spline basis functions, Table S1, and Figures S1 - S6. [file 12859_2024_5636_MOESM1_ESM.docx]

###### Supplemental Information

Spline basis functions:

$$\phi_{0i}\left( t \right)=(t_{i+1}-t_{i})/h_{i}$$

$$\phi_{1i}(t)=(t_{i}-t_{i+1})/h_{i}$$

$$\gamma_{0i}(t)=\left( {\phi_{0i}\left( t \right)}^{3}-\phi_{0i}(t) \right)h_{j}^{2}/6$$

$$\gamma_{1i}(t)=\left( {\phi_{1i}\left( t \right)}^{3}-\phi_{1i}(t) \right)h_{j}^{2}/6$$

Table S1 Number of curve configuration possibilities at 2 - 8 timepoints. Additionally, to 1, 2, 3, or 4 consecutive extrema, two monotone in/decreasing curves and a constant, never changing configuration are possible. Since an extremum is assigned to its nearest knot, a extremum can occur at every knot.

| Extrema count  Knot  count | constant | 0 | 1 | 2 | 3 | 4 | Sum |
| --- | --- | --- | --- | --- | --- | --- | --- |
| 2 | 1 | 2 | 4 | 2 | - | - | **9** |
| 3 | 1 | 2 | 6 | 6 | 2 | - | **17** |
| 4 | 1 | 2 | 8 | 12 | 8 | 2 | **33** |
| 5 | 1 | 2 | 10 | 20 | 20 | 10 | **63** |
| 6 | 1 | 2 | 12 | 30 | 40 | 30 | **115** |
| 7 | 1 | 2 | 14 | 42 | 70 | 70 | **199** |
| 8 | 1 | 2 | 16 | 56 | 112 | 140 | **327** |


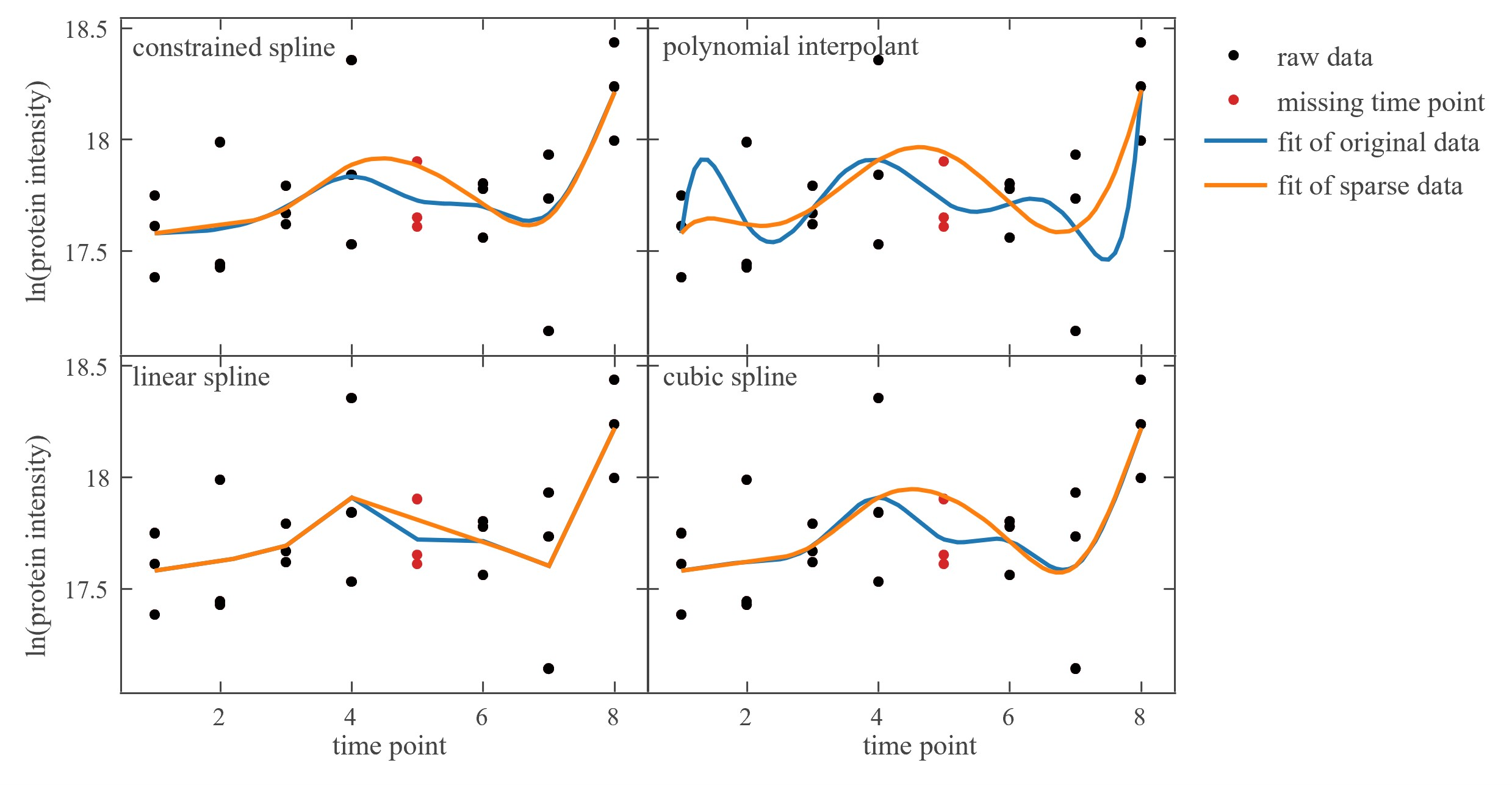


Figure S1. Exemplary leave one out cross validation using four fitting techniques. The blue curves represent the original fit using all available data points. The orange curves represent the fit when all, but the red data points are used. Vertical distances from the blue to the orange curve are measured at time point 5 to assess the robustness of the different fitting techniques.


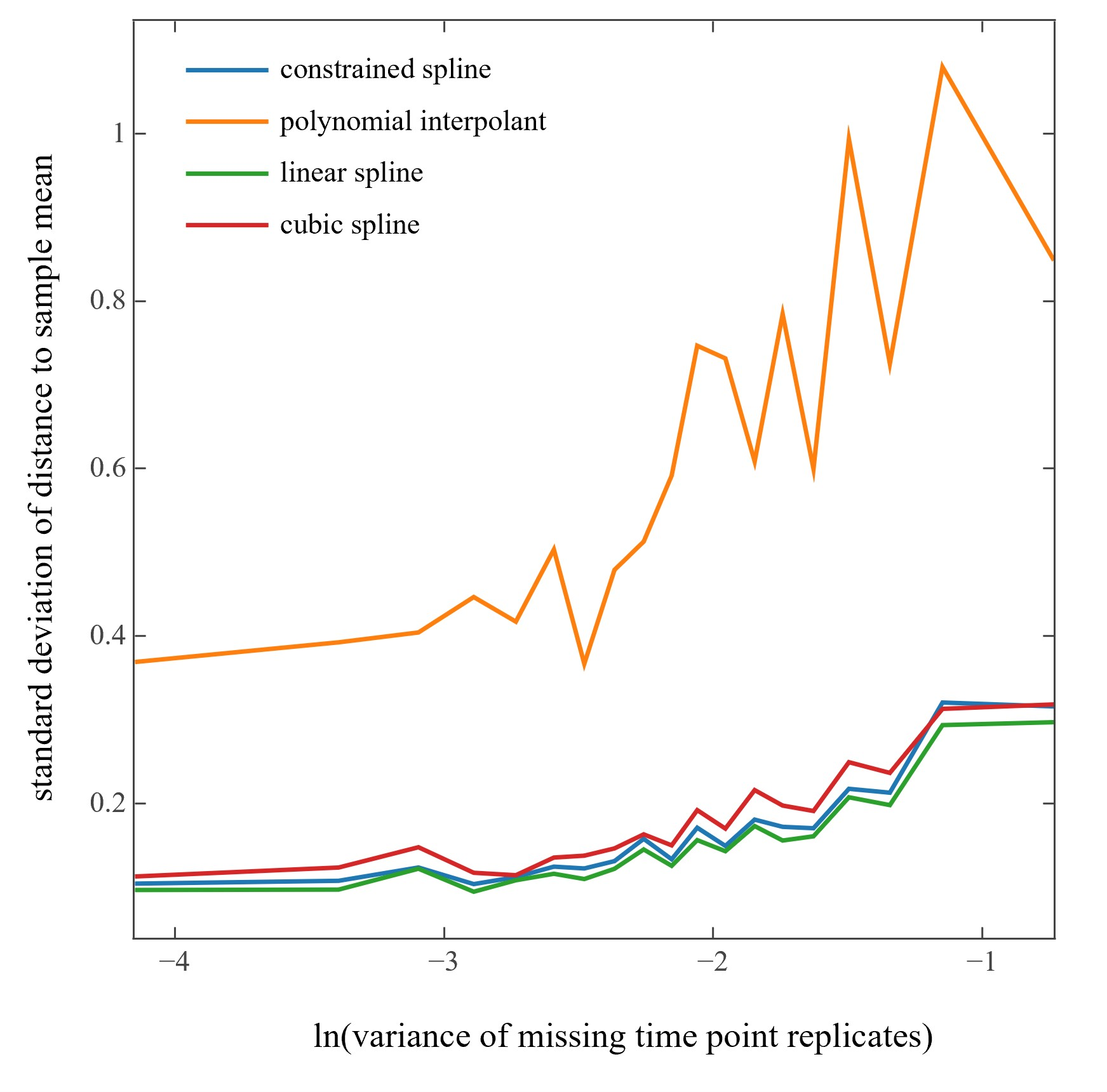


Figure. S2. Fidelity analysis. Four fitting techniques were applied to each protein signal. After deleting every inner time point once, the distance from the sample mean to the prediction using the sparse signal is measured. This data was grouped in 20 equally large bins by the variance of the original time point replicates. The standard deviation of the distances within each bin are calculated and plotted against the average time point variance.


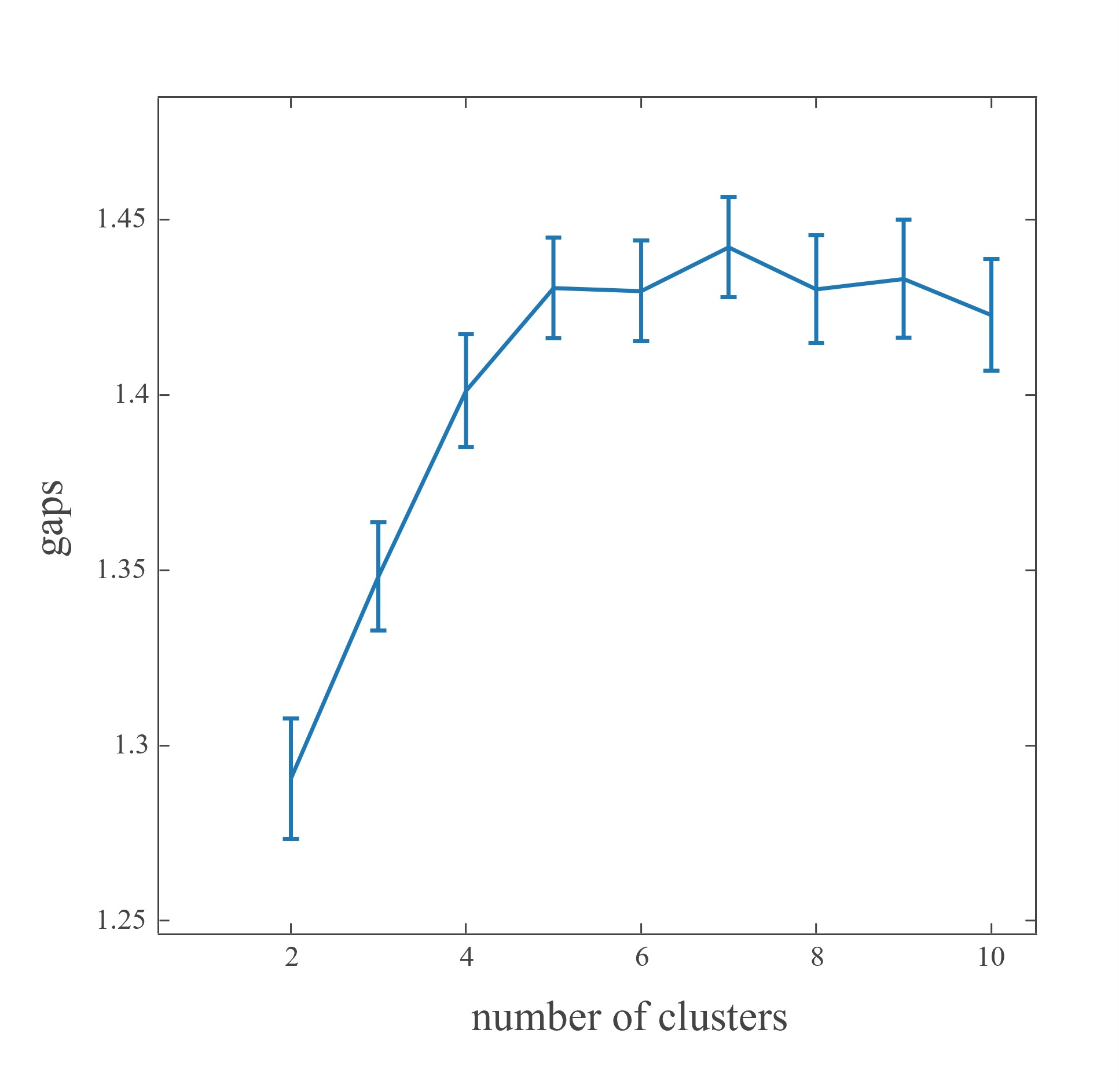


Figure S3. Determination of optimal cluster number via gap statistics. The optimal cluster number is the smallest k such that gap(k)≥ gap(k+1)-s(k+1) where s is the standard deviation of the reference dispersion multiplied by sqrt(1+1/bootstraps). 400 bootstrap iterations where performed. The optimal cluster number was determined to be 5.


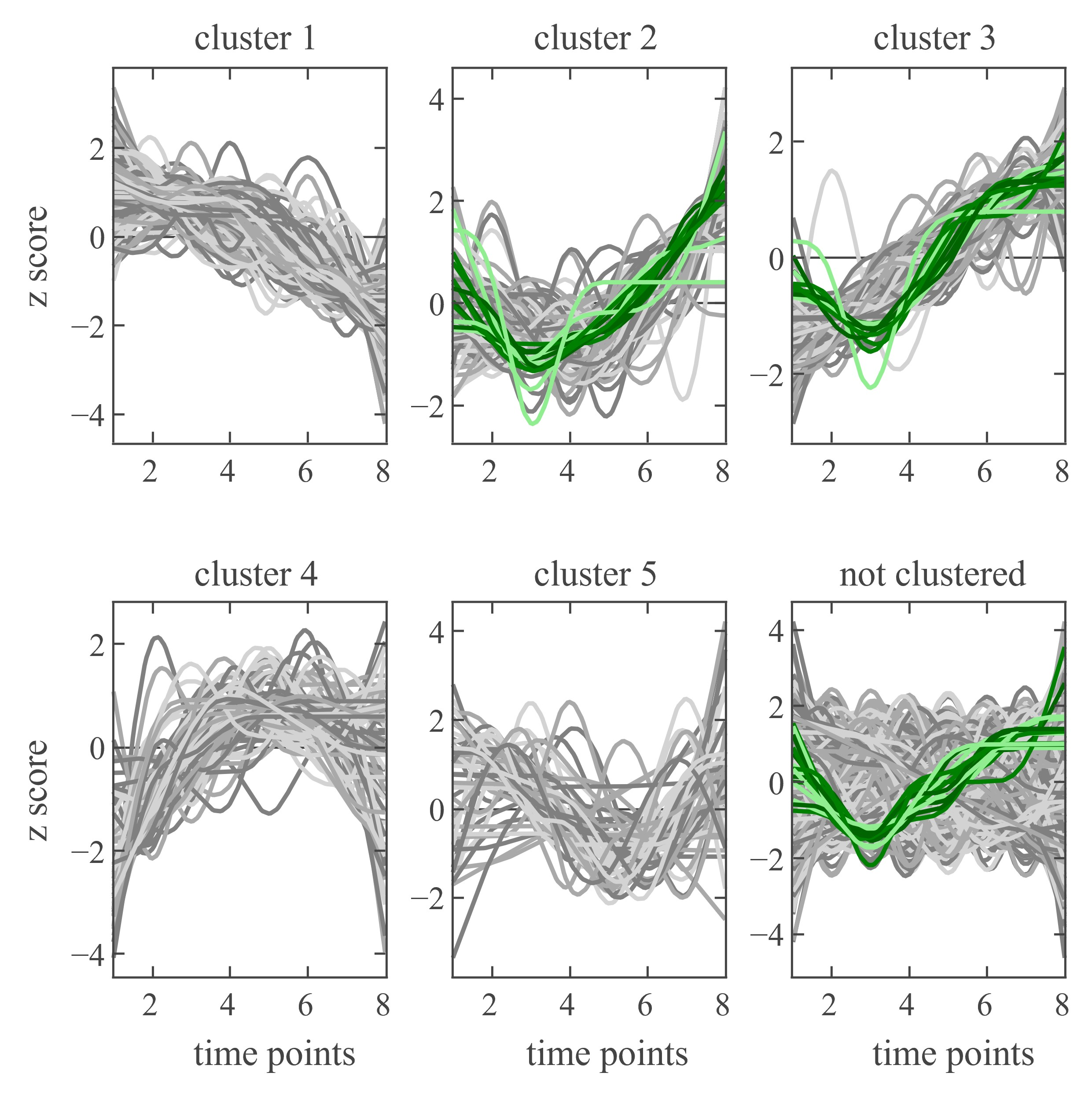


Figure S4. Example of distribution of smoothed protein signals that are member of class “Minimum at TP3” in different clusters.


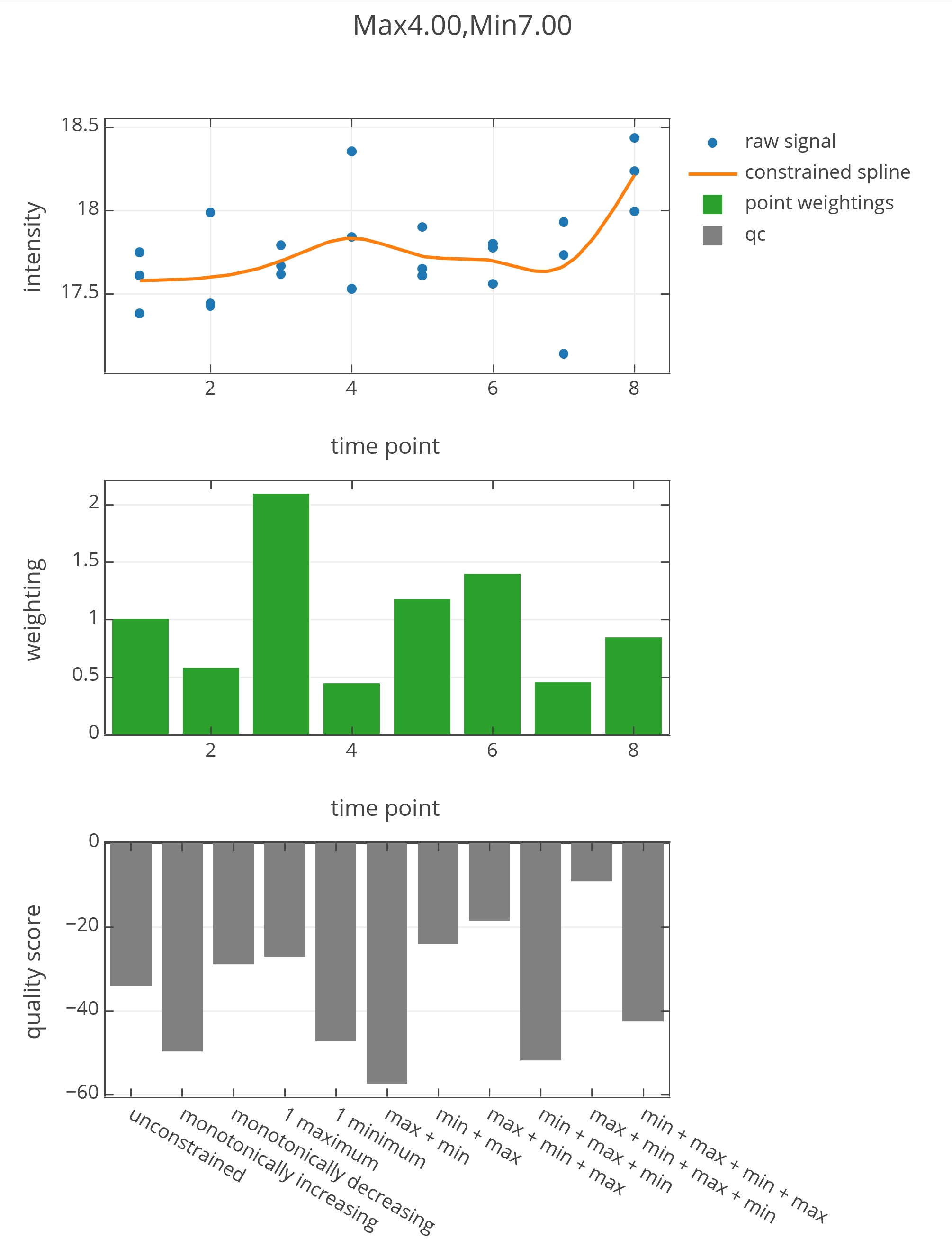


Figure S5 Example of protein-wise result visualization facilitated by the TempClass package available at <https://github.com/CSBiology/TempClass>.


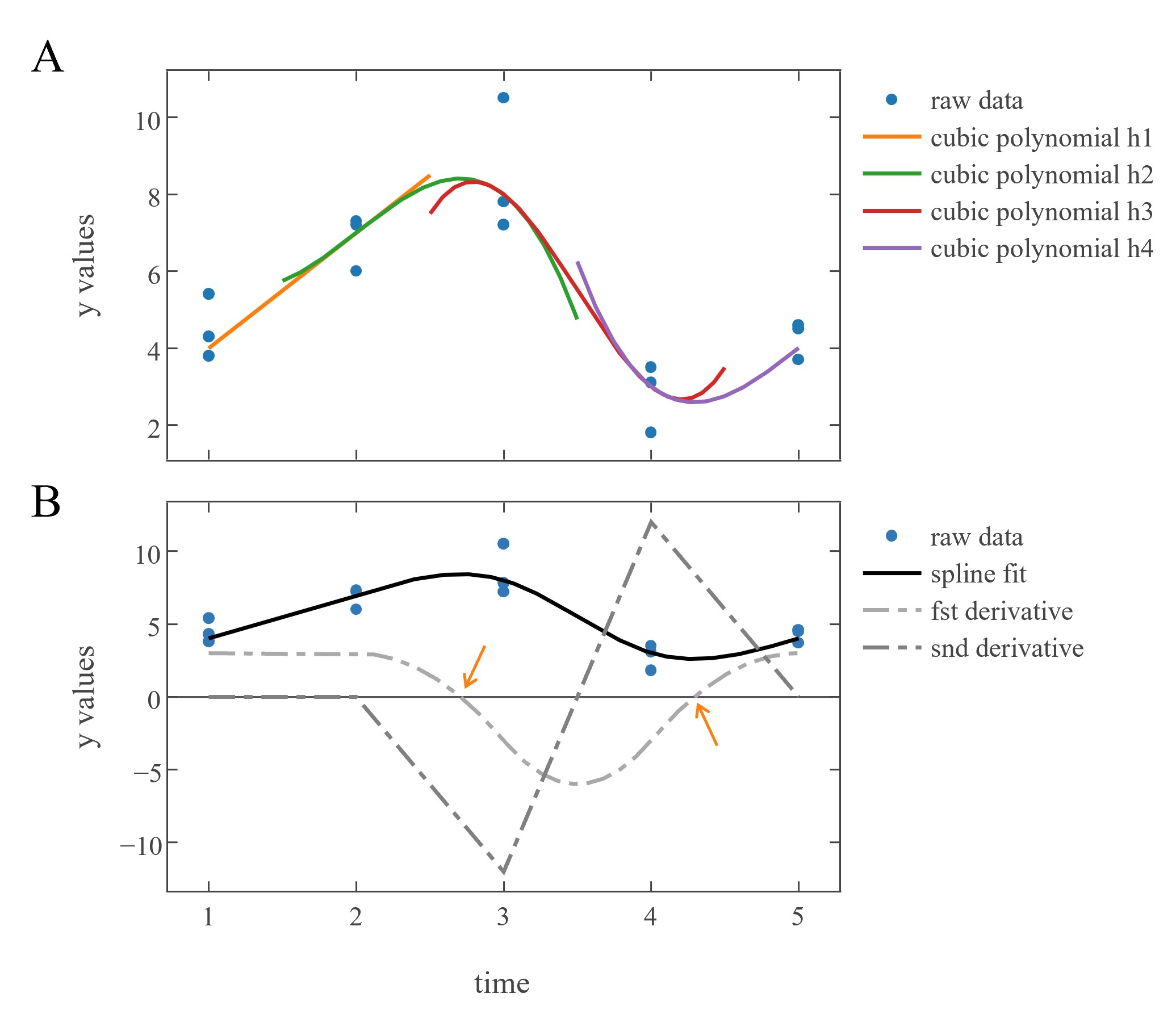
Figure S6. Extrema extraction **A** The constrained smoothing spline of five knots consists of four cubic polynomials that are joined at the knots. **B** From the resulting spline (black) the first and second derivative are determined and visualized (dashed lines). Orange arrows mark time points of zero slope that indicate a maximum if the second derivative is negative or a minimum if the second derivative is positive.
